# Application of Logistic Regression and Decision Tree Models in the Prediction of Activities of Daily Living in Patients with Stroke

**DOI:** 10.1155/2022/9662630

**Published:** 2022-01-28

**Authors:** Qile Zhang, Zheyu Zhang, Xiuqing Huang, Chun Zhou, Jian Xu

**Affiliations:** ^1^Department of Rehabilitation, The Quzhou Affiliated Hospital of Wenzhou Medical University, Quzhou People's Hospital, Quzhou, China; ^2^The Second Clinical Medical College, Zhejiang Chinese Medical University, Hangzhou, China

## Abstract

An improvement in the activities of daily living (ADLs) is significantly related to the quality of life and prognoses of patients with stroke. However, the factors predicting significant improvement in ADL (SI-ADL) have not yet been clarified. Therefore, we sought to identify the key factors affecting SI-ADL in patients with stroke after rehabilitation therapy using both logistic regression modeling and decision tree modeling. We retrospectively collected and analyzed the clinical data of 190 patients with stroke who underwent rehabilitation therapy at our hospital between January 2020 and July 2020. General and rehabilitation therapy data were extracted, and the Barthel index (BI) score was used for outcome assessment. We defined SI-ADL as an improvement in the BI score by 15 points or more during hospitalization. Logistic regression and decision tree models were established to explore the SI-ADL predictors. We then used receiver operating characteristic (ROC) curves to compare the logistic regression and decision tree models. Univariate analysis revealed that compared with the non-SI-ADL group, the SI-ADL group showed a significantly shorter course of stroke, longer hospital stay, and higher rate of receiving occupational and speech therapies (all *P* < 0.05). Binary logistic regression analysis revealed the course of stroke at admission (odds ratio (OR) = 0.986, 95%confidence interval (CI) = 0.979–0.993; *P* < 0.001) and the length of hospital stay (OR = 1.030, 95%CI = 1.013–1.047; *P* =0.001) as the independent predictors of SI-ADL. ROC comparisons revealed no significant differences in the areas under the curves for the logistic regression and decision tree models (0.808 *vs.* 0.831; *z* = 0.977, *P* = 0.329). Both models identified the course of disease at admission and the length of hospital stay as key factors affecting SI-ADL. Early initiation of rehabilitation therapy is of immense importance for improving the ADLs in patients with stroke.

## 1. Introduction

Stroke is a disease with focal neurological deficits caused by sudden cerebral blood circulation abnormalities [1]; it is associated with high mortality and disability rates. Although stroke-associated mortality has decreased with the improvement of medical technology, the number of patients with poststroke motor, sensory, speech, cognitive, psychological, and other dysfunctions has increased sharply [2]. In China, approximately 2 million patients are diagnosed with new-onset stroke every year [3]. Approximately 75% of these stroke survivors have varying degrees of disability; among these, more than 40% are severely disabled [4, 5]. This not only has a marked impact on the activities of daily living (ADLs) of patients but also places a heavy burden on their families.

Improvement in ADLs is significantly related to the quality of life and prognoses of patients with stroke. Previous studies have reported that rehabilitation therapy can improve limb function and ADLs, thereby helping patients return to normal life [6, 7]. In clinical practice, we found that some patients with stroke showed a significant improvement in ADL (SI-ADL) after rehabilitation therapy [8, 9], while other patients only showed a minimal improvement [10]. However, the factors affecting SI-ADL have not yet been clarified.

Therefore, we sought to identify the key factors predicting SI-ADL in patients with stroke after rehabilitation therapy, using both logistic regression modeling and decision tree modeling.

## 2. Materials and Methods

### 2.1. Patient Selection

Between January 2020 and July 2020, 190 patients with stroke underwent rehabilitation therapy at the Department of Rehabilitation of the Quzhou Affiliated Hospital of Wenzhou Medical University. We included patients with stroke, according to the diagnostic criteria adopted by the Fourth National Cerebrovascular Disease Academic Conference of the Chinese Society of Neurology in 1995 [11]. We included patients with stable vital signs or neurological deficit symptoms that no longer progressed after more than 48 hours, who had dysfunction, and who needed rehabilitation intervention. The exclusion criteria were as follows: (1) patients with severe cardiovascular, liver, kidney, digestive, and hematopoietic diseases that may endanger life; (2) patients with serious mental disorders; (3) patients with newly developed intracranial lesions or further aggravation of neurological deficits during hospitalization; (4) patients who refused continued rehabilitation; and (5) patients with incomplete clinical data acquired during hospitalization. We extracted data on demographics (age and sex), medical history, final diagnosis, course of stroke at admission, with or without rehabilitation therapy before admission, length of hospital stay, laboratory test results at admission, and medications used during hospitalization.

This study was approved by the human ethics committee of the Quzhou Affiliated Hospital of the Wenzhou Medical University (LS2018023). Written informed consent was obtained at the time of admission. The clinical investigation was conducted in accordance with the principles of the Declaration of Helsinki.

### 2.2. Rehabilitation Therapies

All patients received exercise therapy and physical factor treatment. In addition, patients received one or more treatments specific to their dysfunctions. Exercise therapy included proper limb positioning, joint range-of-motion training, muscle strength training, turnover, transfer training, bridge exercise, sitting and standing balance training, and walking and up-and-down stair training, among others. Each treatment session lasted for 40 min and was conducted once a day.

Physical factor treatment included neuromuscular electrical stimulation of the hemiplegic side. Different electrical stimulation sites were selected according to the patients' conditions. The commonly used stimulation sites included the deltoid, triceps brachii, extensor carpi longus radialis, quadriceps femoris, and tibialis anterior muscles. Each session lasted for 20 min and was conducted once a day.

Occupational therapy included training in shoulder antexion, abduction, elbow extension, forearm rotation, wrist extension, and finger flexion and extension movements. It also included upper limb virtual games and task-oriented training (such as washing and dressing). Each treatment session lasted for 40 min and was conducted once a day.

For speech therapy, the Schuell stimulation method was adopted to conduct progressive training in audiovisual understanding, retelling, oral expression, reading, and writing in a one-to-one manner. Each treatment session lasted for 40 min and was conducted once a day.

Cognitive therapy included computer-assisted targeted training in attention, orientation, visual space, executive ability, memory, and logical thinking. Each treatment session lasted for 40 min and was conducted once a day.

Swallowing therapy included guided lip exercises, ice stimulation of the oral and throat muscles, supraglottic swallowing, forced swallowing, empty swallowing, nodding swallowing, Mendelsohn swallowing and shaker manipulation, free drinking water training, and limiting the amount of one mouthful. Each treatment session lasted for 40 min and was conducted once a day.

Acupuncture treatment included stimulation of the acupoints in the Yangming meridian of the upper and lower limbs during the period of soft paralysis. For spasms, the principle of “taking acupoints by antagonistic muscles” was adopted. The acupoints that were often stimulated included the hand Sanli, Waiguan, Hegu, Jianyu, Bige, Yanglingquan, Zusanli, Jiexi, Weizhong, and knee Yangguan. Acupuncture was administered using 1.5–2.0-inch No. 30 acupuncture needles. The needles were inserted for 20 min, once a day.

Respiratory therapy included guided chest-expansion exercises, abdominal breathing training, and respiratory function improvement through the use of an incentive spirometer. For patients with foot drop and varus affecting the walking function, an orthosis was employed. We used a German Ottobock 50s1 ankle-foot orthosis for walking training.

For patients with unrelieved poststroke shoulder pain after manipulation and physical factor treatments, the lesion site on the shoulder was examined under the guidance of color Doppler ultrasound, and local injections were administered. These comprised a 3 mL lidocaine hydrochloride injection, 1 mL compound betamethasone injection, and 0.9% sodium chloride injection. These injections were administered only once in selected patients.

### 2.3. Outcome Evaluation

Data on the Barthel index (BI) scores at admission and discharge were extracted from the records. The BI scale assesses 10 ADLs, namely, feeding, bathing, grooming, dressing, bowels, bladder, toilet use, transfers, mobility, and stairs. Each item was scored 5, 10, or 15 points, and the total scores ranged from 0 to 100. A BI score of 60 points was chosen as the cut-off: a score ≥ 60 points indicated that the patient lived mostly or completely independently, while a score < 60 points indicated that the patient lived mostly or in complete dependence on the care of others [12]. In this study, SI-ADL was defined by an at least 15-point increase in the BI score at discharge. No significant improvement in the ADL (NSI-ADL) was defined by a less than 15-point increase in the BI score.

### 2.4. Development of the Logistic Regression Model and the Decision Tree Model

A binary logistic regression analysis model was established by taking variables with *P* < 0.05 in the univariate analysis as the independent variables and SI-ADL as the dependent variable.

Variables with *P* < 0.05 in the univariate analysis were further analyzed to develop the decision tree model. The model was established using the classification and regression tree method; the decision tree analysis was performed using the SPSS software (version 22.0). The decision tree grew “branches” by significance testing, with a split occurring at *α* = 0.05. The time limit specified that the minimum sample size of the parent node was 20, and the minimum sample size of the offspring node was 5. If the sample size on the node failed to meet this requirement, the node was considered a terminal node and was not segmented.

### 2.5. Statistical Analysis

The SPSS 22.0 software (IBM) was used for data analysis. Continuous data are presented as frequencies and percentages. The two groups of measurement data are presented as means ± standard deviations or as medians (interquartile ranges, IQRs). The *χ*^2^ test was used for the comparison of categorical variables, while the *t*-test or the Mann–Whitney nonparametric test was used for the comparison of continuous variables. Spearman correlation analysis was conducted to identify the correlations between the variables. Baseline variables with *P* < 0.05 in the univariate analysis were used to develop the binary logistic regression analysis model and the decision tree model, separately. Implementing the Delong method, receiver operating characteristic (ROC) curves obtained from the logistic regression and decision tree models were then compared using the MedCalc 15.0 software (MedCalc Software, Mariakerke, Belgium). Statistical significance was set at *P* < 0.05.

## 3. Results

### 3.1. Baseline Characteristics

**Figure 1:**
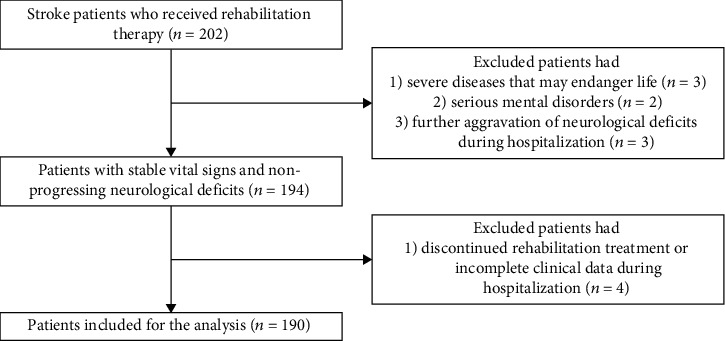
Study flowchart.

Overall, 190 patients with stroke met the criteria for inclusion in our study (Figure 1); of these, 109 (57.4%) were diagnosed with cerebral infarction and 66 (34.7%) were women. The median age was 59 years (IQR: 48–70 years). The median course of stroke at admission was 49 days (IQR: 21–97 days), and the median length of hospital stay was 23 days (IQR: 13–39 days). A total of 110 patients (57.9%) had received rehabilitation therapy before admission. The median BI score of all patients was 45 (IQR: 20–66) at admission and 60 (IQR: 40–80) at discharge. Overall, 94 patients (49.5%) had a BI score ≥ 60 at discharge and 80 patients (42.1%) had SI-ADL at discharge.

### 3.2. Relationship between Rehabilitation Therapy and BI

Compared with patients who did not receive rehabilitation therapy before admission, those who had received rehabilitation therapy before admission had a higher BI score (45 *vs.* 35 points, *Z* = −2.132, *P* = 0.033) and a longer course of stroke (78 *vs.* 20 days, *t* = 7.372, *P* < 0.001); they also comprised a lower proportion of patients with cerebral infarction (50.9% *vs.* 66.3%, *χ*^2^ = 4.457, *P* = 0.035).

The BI scores at discharge of both patients with and without prior rehabilitation therapy had significant improvement when compared with the corresponding BI scores at admission (all *P* < 0.001); however, the BI scores at discharge themselves did not differ significantly between these two groups (with and without prior rehabilitation therapy: 60 *vs.* 65 points, *Z* = −0.468, *P* = 0.639). At discharge, there was no significant difference in the proportion of patients with SI-ADL between these two groups (with and without prior rehabilitation therapy: 38.2% *vs.* 47.5%, *χ*^2^ = 1.650, *P* = 0.199).

During hospitalization, all patients received exercise and physical therapies. Therefore, exercise therapy and physical therapy were not included in the calculation of the rehabilitation therapy types. Spearman's correlation analysis revealed that the number of rehabilitation therapies received by patients during hospitalization was positively correlated with the course of stroke at admission (*ρ* = 0.197, *P* = 0.006) and the length of hospital stay (*ρ* = 0.277, *P* < 0.001) and negatively correlated with a cerebral infarction diagnosis (*ρ* = −0.248, *P* = 0.001). There was no correlation between the number of rehabilitation therapies and SI-ADL (*ρ* = 0.088, *P* = 0.288).

There were no significant differences in the lengths of hospital stay between the group with a BI score < 60 at discharge and the group with a BI score ≥ 60 at discharge (median: 21 *vs.* 26, *Z* = −0.799, *P* = 0.424). In addition, the group with a BI score ≥ 60 at discharge received significantly fewer rehabilitation therapy types as compared with the group with a BI score < 60 at discharge (1 *vs.* 2, *Z* = −4.727, *P* < 0.001). The group with a BI score ≥ 60 at discharge received lesser speech, cognitive, swallowing, and respiratory therapies (all *P* ≤ 0.001; Table 1). Additionally, patients who received speech, cognitive, swallowing, and respiratory therapies had significantly lower BI scores than those who did not receive these four rehabilitation therapies (all *P* < 0.001; see Supplementary table I). However, multivariate regression analysis revealed that none of these four therapies were risk factors for achieving a BI score ≥ 60 at discharge (see Supplementary Tables II and III).

### 3.3. Factors Associated with SI-ADL

Univariate analysis revealed that compared with patients with NSI-ADL, patients with SI-ADL had a shorter course of stroke at admission and a longer length of hospital stay and also comprised a higher proportion of those receiving occupational and speech therapies (all *P* < 0.05). There was no significant difference in the proportion of patients receiving cognitive, respiratory, and swallowing therapies between the NSI-ADL and SI-ADL groups (all *P* > 0.05; Table 2).

### 3.4. Characteristics of Patients with SI-ADL Based on the Decision Tree Model

**Figure 2:**
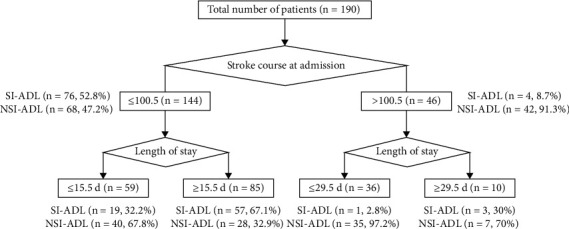
The SI-ADL decision tree model. The first decision node indicates the course of the stroke at admission, followed by the length of hospital stay.

The baseline variables with *P* < 0.05 in the univariate analysis were also used to develop the decision tree model to predict SI-ADL (Figure 2). The results showed that among the patients with a course of disease ≤ 100.5 days at admission, 52.8% had SI-ADL. Among the patients with a length of hospital stay > 15.5 days, 67.1% achieved SI-ADL. Only 32.2% of the patients with a length of hospital stay ≤ 15.5 days achieved SI-ADL. Among the patients with a course of disease > 100.5 days at admission, only 8.7% achieved SI-ADL after hospitalization. Among the patients whose length of hospital stay was >29.5 days, 30.0% achieved SI-ADL. Only 2.8% of the patients with a length of hospital stay < 29.5 days achieved SI-ADL.

### 3.5. Comparison of the Logistic Regression Model and the Decision Tree Model

**Figure 3:**
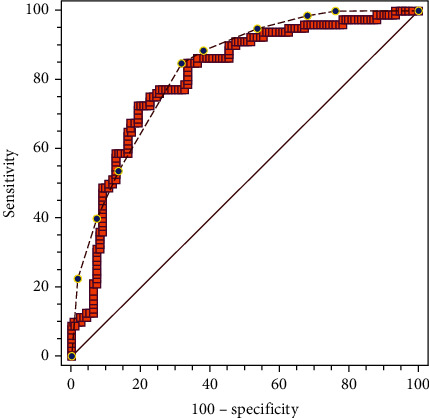
Comparison of the receiver operating characteristic curves between the two models. The red line with blue dots is the curve of the decision tree model, while the orange line is the curve of the logistic regression model. The area under the curve is 0.808 for the logistic regression model and 0.831 for the decision tree model. There are no significant differences between the two models.

For the logistic regression model, the area under the curve (AUC) was 0.808 (95%CI = 0.744–0.861, *P* = 0.032). For the decision tree model, the AUC was 0.831 (95%CI = 0.770–0.881, *P* = 0.029). Comparison of the ROC curves of the logistic regression and the decision tree models revealed no significant differences in the AUCs between the two (*z* = 0.977, *P* = 0.329; Figure 3).

## 4. Discussion

We investigated the factors that predict SI-ADL after rehabilitation therapy in patients with stroke. Both the logistic regression model and the decision tree model confirmed that the course of disease at admission and the length of hospital stay were the key factors affecting SI-ADL.

Previous studies have shown that the ADL level of patients with stroke at admission is positively correlated with the ADL level at discharge, suggesting that the higher the degree of functional independence at baseline, the better the effect of the rehabilitation therapy. However, in contrast to previous research, our study found that although the BI score (at admission) of patients who had received rehabilitation therapy before admission was higher than the score of those who had not, there were no significant differences in the BI scores at discharge between the two groups after receiving the rehabilitation therapy. This suggested that the plasticity of functional recovery in patients who had previously received rehabilitation therapy was relatively low. This may be because patients who have received rehabilitation therapy before admission usually have a longer course of the disease. Therefore, when they receive rehabilitation therapies for the second time, their sensitivities to these therapies are much lower than those of patients who have not received rehabilitation therapy previously. Li and Zhong [13] categorized 45 patients with stroke into three groups according to the disease course. Patients in whom the disease course was <1 month, 1–6 months, and >6 months were included in the first, second, and third groups, respectively. The ADL scores were evaluated before and after the comprehensive rehabilitation therapy. They found that the ADL scores of groups 1 and 2 were significantly improved after the rehabilitation therapy, but there was no significant increase in the ADLs in group 3 after rehabilitation. This study showed that the effect of rehabilitation therapy varies significantly at different stages after stroke onset. Moreover, patients who received rehabilitation therapy before admission were mostly patients with intracerebral hemorrhage; previous studies have shown that such patients are likely to have more serious sensory, motor, and cognitive impairments. Therefore, they require additional rehabilitation therapy, and the rehabilitation process is more difficult [14, 15]. Moreover, the degree of recovery of ADLs in patients with intracerebral hemorrhage is time-dependent; i.e., the later the rehabilitation intervention commences, the more difficult is the recovery [16]. Therefore, in patients who have already received rehabilitation therapy, the effect of the second rehabilitation therapy largely depends on the timing of the treatment. If the course of the disease is too long, even if they receive rehabilitation therapy again, the treatment effect may still be unsatisfactory.

In addition, a large number of previous studies have shown that the types of rehabilitation therapies are positively correlated with an increase in ADLs [17–20]. Zhang and Zhang [21] divided 160 patients with acute stroke into two groups: 80 patients in the control group were treated with conventional medical drugs, while 80 patients in the study group were treated with comprehensive rehabilitation therapy (such as exercise therapy, acupuncture, and traditional Chinese medicine). After 4 weeks, the limb motor ability and the BI scores of the patients in the study group were higher than those of the patients in the control group. Therefore, it is considered that comprehensive rehabilitation therapy can reduce the neurological deficit of patients, promote functional recovery of hemiplegic limbs, and improve ADLs. However, our study showed that patients who received more types of rehabilitation therapy did not have improved ADLs as compared with patients who received fewer types of rehabilitation therapies. This may be because the more the rehabilitation therapy administered to a patient, the more severe is the loss of basic neurological function, the longer is the course of the disease, and the longer is the hospital stay. It is worth noting that the condition of patients receiving comprehensive rehabilitation therapy still improved after treatment, and the two groups of patients had similar rehabilitation outcomes at discharge; this suggests that it is still valuable to provide comprehensive rehabilitation therapy and appropriately prolong the treatment time for patients with a serious condition and a long course of the disease. However, in the short term, it may not be possible to achieve a better recovery effect than in patients with milder symptoms.

Based on univariate analysis, it was clear that the course of the disease and a previous implementation of rehabilitation therapy at admission had an impact on the improvement of ADLs. In addition, through a binary logistic regression analysis, we showed that the course of the disease at admission and the length of hospital stay are the key factors for significant improvement in the ADLs; i.e., early rehabilitation intervention for patients with stroke and prolonged duration of rehabilitation treatment can improve the ADLs.

However, because logistic regression cannot quantify the variables that are meaningful for classification and the value of guiding the patients' treatment strategies in the clinic is limited, we further performed a decision tree analysis. The decision tree model is a reliable and effective analysis tool that can build an intuitive and understandable tree structure, quantifying the specific variables of certain prediction results and providing a basis for decision-making. Comparison of the ROC curves between the logistic regression model and the decision-tree model confirmed that the predictive value of the decision-tree model was not inferior to that of the logistic regression model; the decision tree could be used to formulate individualized rehabilitation strategies for patients with stroke.

The first consideration was the course of the disease at admission. For patients in whom the course of the disease at admission was less than 100.5 days, the probability of SI-ADL after hospitalization for rehabilitation therapy for more than 2 weeks was 67.1%. However, the probability of SI-ADL was significantly lower in patients with a length of hospital stay of shorter than 2 weeks (only 32.2%). For patients in whom the course of the disease exceeded 100.5 days at admission, the probability of an SI-ADL was approximately 30.0% for those who were hospitalized for more than 1 month and only 2.8% for those who had been hospitalized for less than 1 month. This suggests that for patients with a long course of the disease (more than 3 months), the effect of short-term rehabilitation therapy is minimal. On the other hand, long-term inpatient rehabilitation therapy can significantly improve ADLs. Although the guidelines set the time of rehabilitation therapy for patients with stroke to within 48 hours to 2 weeks after the condition is stable, some studies have pointed out that the golden period of rehabilitation therapy is within 3 months after the stroke [22]. This is almost consistent with the 100.5 days of the course node automatically defined in the decision-tree analysis in this study. Ballester et al. [23] also found that with a longer course of the disease, the slow rehabilitation effect is due to the gradual decline in the patients' sensitivity to the treatment. For more than a year after the stroke, the patients' nerves still had some plasticity. By formulating accurate plans and adopting continuous individualized and progressive rehabilitation therapy schemes, the sensitivity to treatment can be increased and ADLs can still be improved.

The inability to implement and failure to implement rehabilitation early in many areas in China are some of the reasons for the nationwide high morality and mortality rates in patients with stroke; this has led to a huge socioeconomic burden [24]. Based on our study, although patients with late initiation of rehabilitation still benefited from adequate rehabilitation, those who had not received previous rehabilitation therapy had a lower BI score at admission. Those with lower BI scores required more types of rehabilitation during hospitalization, which resulted in an increased cost of rehabilitation. Furthermore, the shorter the stroke course, the shorter the time required for rehabilitation. Therefore, early initiation and adequate rehabilitation after stroke may be an important step for reducing the socioeconomic burdens on patients with stroke. However, early initiation of rehabilitation is limited by several barriers [25]. Tam et al. [26] used a rapid access outpatient stroke rehabilitation program for providing rehabilitation. This approach could alleviate problems such as rehabilitation ward unavailability and the inability to treat patients in the hospital for a long period. It could also improve patient compliance with rehabilitation and help both the doctors and patients choose the timing of rehabilitation initiation and treatment delivery in a flexible manner.

The limitations of this study were as follows. First, this was a retrospective single-center study with a small sample size, and selection bias may have occurred. In the future, we will perform prospective studies and expand the sample sizes to obtain more accurate results. Second, we did not conduct a long-term follow-up on the functional outcomes of the patients after discharge, because the description of the outcomes of patients with stroke generally includes the functional status, length of stay, and destination after discharge [27]. The length of stay is largely affected by the patient's economic status, medical insurance system, and other factors. In China, the postdischarge destination of patients with stroke is typically their homes, rather than nursing homes and care institutions for older patients. Thus, the length of hospital stay would be prolonged; this limits postdischarge destination as an outcome evaluation index. The functional status of the patients at discharge can avoid the influence of social factors; therefore, it is the most reliable predictor of patient outcomes after stroke [22].

## 5. Conclusions

Early initiation of rehabilitation and sustained rehabilitation therapy plays a key role in improving ADLs. Therefore, providing continuous and sustained rehabilitation therapy to patients with stroke, as early as possible, will help improve the efficiency of the rehabilitation therapy. This can significantly improve the quality of life of these patients.

## Figures and Tables

**Table 1:** Comparison of the rehabilitation therapies between patients with a BI score < 60 and ≥60 at discharge.

	BI score < 60 at discharge	BI score ≥ 60 at discharge	Test value	*P* value
Occupational therapy	87 (90.6)	87 (92.6)	*χ* ^2^ = 0.229	0.632
Speech therapy	61 (63.5)	40 (42.6)	*χ* ^2^ = 8.402	0.004
Cognitive therapy	49 (51)	21 (22.3)	*χ* ^2^ = 16.814	<0.001
Swallowing therapy	40 (41.7)	15 (16)	*χ* ^2^ = 15.263	<0.001
Acupuncture treatment	18 (18.8)	12 (12.8)	*χ* ^2^ = 1.279	0.258
Respiratory therapy	34 (35.4)	6 (6.4)	*χ* ^2^ = 24.088	<0.001
Configuration of orthosis	11 (11.5)	5 (5.3)	*χ* ^2^ = 2.321	0.128
Steroid injection	6 (6.3)	4 (4.3)	*χ* ^2^ = 0.379	0.538
Numbers of rehabilitation therapies, median (IQR)	2 (1–4)	1 (0–2)	*Z* = −5.031	<0.001

BI: Barthel index; IQR: interquartile range; WBC: white blood cell; LDL: low-density lipoprotein.

**Table 2:** Baseline characteristics between SI-ADL and NSI-ADL groups.

	Non-SI-ADL (*n* = 110)	SI-ADL (*n* = 80)	Test value	*P* value
Age (years), median (IQR)	60 (49–70)	58 (46–71)	*Z* = −0.421	0.674
Female, *n* (%)	38 (34.6)	28 (35.0)	*χ* ^2^ = 0.004	0.948
Cerebral infarction, *n* (%)	66 (60.0)	43 (53.8)	*χ* ^2^ = 0.740	0.390
Course of disease at admission (days) (IQR)	61 (29–142)	31 (10–65)	*Z* = −4.048	<0.001
Past medical history, *n* (%)				
Hypertension	86 (78.2)	59 (73.8)	*χ* ^2^ = 0.503	0.478
Diabetes	38 (34.6)	18 (22.5)	*χ* ^2^ = 3.233	0.072
Coronary heart disease	13 (11.8)	1 (1.3)	*χ* ^2^ = 7.579	0.006
Atrial fibrillation	10 (9.1)	4 (5.0)	*χ* ^2^ = 1.136	0.287
Stroke	14 (12.7)	5 (6.3)	*χ* ^2^ = 2.159	0.142
Length of stay (days) (IQR)	14 (13–20)	30 (15–43)	*Z* = −4.707	<0.001
Systolic blood pressure at admission (mmHg) ($\bar{x\;}\pm s$)	130.68 ± 16.948	131.75 ± 19.255	*t* = 0.400	0.690
Diastolic blood pressure at admission (mmHg) ($\bar{x}\pm s$)	79.64 ± 12.336	79.65 ± 13.677	*t* = 0.001	1.000
WBC count at admission (10^9^/L) ($\bar{x}\pm s$)	6.61 ± 2.418	6.70 ± 2.865	*t* = 0.224	0.823
LDL level at admission, (mmol/L) (IQR)	2.09 (1.60–2.54)	2.09 (1.68–2.97)	*Z* = 1.790	0.075
BI score at admission (IQR)	48 (28–75)	35 (20–55)	*Z* = −2.609	0.009
BI score at discharge (IQR)	50 (30–80)	65 (50–84)	*Z* = −2.451	0.014
Antihypertensive therapy, *n* (%)	70 (63.6)	46 (57.5)	*χ* ^2^ = 0.733	0.392
Hypoglycemic therapy, *n* (%)	33 (30.0)	20 (25.0)	*χ* ^2^ = 0.576	0.448
Occupational therapy, *n* (%)	97 (81.2)	77 (96.3)	*χ* ^2^ = 3.909	0.048
Speech therapy, *n* (%)	49 (44.6)	52 (65.0)	*χ* ^2^ = 7.782	0.005
Cognitive therapy, *n* (%)	37 (33.6)	33 (41.3)	*χ* ^2^ = 1.154	0.283
Swallowing therapy, *n* (%)	34 (30.9)	21 (26.3)	*χ* ^2^ = 0.489	0.484
Acupuncture treatment, *n* (%)	16 (14.6)	14 (17.5)	*χ* ^2^ = 0.304	0.581
Respiratory therapy, *n* (%)	24 (21.8)	16 (20.0)	*χ* ^2^ = 0.092	0.761
Configuration of orthosis, *n* (%)	6 (5.5)	1 (12.5)	*χ* ^2^ = 2.981	0.084
Steroid injection, *n* (%)	7 (6.4)	3 (3.8)	*χ* ^2^ = 0.635	0.426
Numbers of rehabilitation therapies, median (IQR)	1 (0–3)	2 (1–3)	*Z* = −1.680	0.093

IQR: interquartile range; WBC: white blood cell; LDL: low-density lipoprotein; BI: Barthel index. The baseline variables with *P* < 0.05 in the univariate analysis were included in the binary logistic regression analysis. The results showed that the stroke course at admission (odds ratio (OR) = 0.986, 95%confidence interval (CI) = 0.979–0.993, *P* < 0.001) and the length of hospital stay (OR = 1.030, 95%CI = 1.013–1.047, *P* = 0.001) were the significant predictors of SI-ADL, while occupational therapy (OR = 3.737, 95%CI = 0.930–15.017, *P* = 0.063) and speech therapy (OR = 1.625, 95%CI = 0.812–3.252, *P* = 0.170) were not.

## Data Availability

The original data used to support the findings of this study are available from the corresponding author upon request.

## Supplementary Materials

Supplementary Materials Supplementary table I: comparison of the BI scores at admission between patients with and without rehabilitation therapy. Supplementary table II: univariate comparisons between BI scores < 60 and ≥60 at discharge. Supplementary table III: multivariate regression analysis for BI scores ≥ 60 at discharge.
