# Supplementary material for: Application of Logistic Regression and Decision Tree Models in the Prediction of Activities of Daily Living in Patients with Stroke
Source: Neural Plast. 2022 Jan 28;2022:9662630. doi: 10.1155/2022/9662630 (PMC8816537; doi:10.1155/2022/9662630)
Supplement: Supplementary Materials — Supplementary table I: comparison of the BI scores at admission between patients with and without rehabilitation therapy. Supplementary table II: univariate comparisons between BI scores < 60 and ≥60 at discharge. Supplementary table III: multivariate regression analysis for BI scores ≥ 60 at discharge. [file 9662630.f1.docx]

**Supplementary table I. Comparison of BI score at admission between patients with and without rehabilitation therapy**

| Rehabilitation therapy | | BI score at admission, median (IQR) | Z value^*^ | *P* value |
| --- | --- | --- | --- | --- |
| Speech therapy | No | 55 (35-72) | -5.237 | <0.001 |
|  | Yes | 30 (12-55) |  |  |
| Cognitive therapy | No | 50 (30-70) | -4.867 | <0.001 |
|  | Yes | 25 (10-50) |  |  |
| Swallowing therapy | No | 50 (30-70) | -5.470 | <0.001 |
|  | Yes | 20 (5-50) |  |  |
| Respiratory therapy | No | 50 (30-70) | -6.303 | <0.001 |
|  | Yes | 15 (1-29) |  |  |

*, test value of Mann-Whitney nonparametric test. IQR, inter quartile range.

**Supplementary table II. Univariate comparisons between BI score <60 and ≥60 at discharge.**

|  | BI score <60 at discharge | BI score ≥ 60 at discharge | test value | *P* value |
| --- | --- | --- | --- | --- |
| Age, years, median (IQR) | 63 (50-73) | 54 (44-66) | Z = -2.868 | 0.004 |
|  |  |  |  |  |
| Female, n(%) | 36 (62.5) | 30 (31.9) | χ^2^ = 0.654 | 0.419 |
| Cerebral infarction, n(%) | 46 (47.9) | 63 (67) | χ^2^ = 7.088 | 0.008 |
| Course of disease before admission, days (IQR) | 49 (24-92) | 45 (19-116) | Z = -0.021 | 0.983 |
| Medical history, n(%) |  |  |  |  |
| Hypertension | 76 (79.2) | 69 (73.4) | χ^2^ = 0.873 | 0.350 |
| Diabetes | 20 (20.8) | 36 (38.3) | χ^2^ = 6.969 | 0.008 |
| Coronary heart disease | 9 (9.4) | 5 (5.3) | χ^2^ = 1.145 | 0.285 |
| Atrial fibrillation | 10 (10.4) | 4 (4.3) | χ^2^ = 2.642 | 0.104 |
| Stroke | 11 (11.5) | 8 (8.5) | χ^2^ = 0.459 | 0.498 |
| Length of stay, days, median (IQR) | 26 (14-40) | 21 (13-38) | Z = -0.799 | 0.424 |
| Systolic blood pressure at admission, mmHg ($\bar{x}$*±s*) | 130.7±18.9 | 131.5±17.0 | t = -0.313 | 0.754 |
| Diastolic blood pressure at admission, mmHg ($\bar{x}$*±s*) | 80.1±13.5 | 79.2±12.3 | t = 0.462 | 0.645 |
| WBC at admission, 10^9^/L ($\bar{x}$*±s*) | 7.0±2.5 | 6.3±2.6 | t = 1.784 | 0.076 |
| LDL at admission, mmol/L ($\bar{x}$*±s*) | 2.4±0.8 | 2.0±0.7 | t = 3.220 | 0.002 |
| BI score at admission, median (IQR) | 25 (10-35) | 67 (55-80) | Z = -10.111 | <0.001 |
| Antihypertensive therapy, n(%) | 58 (60.4) | 58 (61.7) | χ^2^ = 0.033 | 0.856 |
| Hypoglycemic therapy, n(%) | 20 (20.8) | 33 (35.1) | χ^2^ = 4.810 | 0.028 |
| Rehabilitation therapy, n(%) |  |  |  |  |
| Occupational therapy | 87 (90.6) | 87 (92.6) | χ^2^ = 0.229 | 0.632 |
| Speech therapy | 61 (63.5) | 40 (42.6) | χ^2^ = 8.402 | 0.004 |
| Cognitive therapy | 49 (51) | 21 (22.3) | χ^2^ = 16.814 | <0.001 |
| Swallowing therapy | 40 (41.7) | 15 (16) | χ^2^ = 15.263 | <0.001 |
| Acupuncture treatment | 18 (18.8) | 12 (12.8) | χ^2^ = 1.279 | 0.258 |
| Respiratory therapy | 34 (35.4) | 6 (6.4) | χ^2^ = 24.088 | <0.001 |
| Configuration of orthosis | 11 (11.5) | 5 (5.3) | χ^2^ = 2.321 | 0.128 |
| Steroid injection | 6 (6.3) | 4 (4.3) | χ^2^ = 0.379 | 0.538 |
| Numbers of rehabilitation therapy, median (IQR) | 2 (1-4) | 1 (0-2) | Z = -5.031 | <0.001 |

BI, Barthel index; IQR, inter quartile ratio; WBC, white blood cell; LDL, low density lipoprotein.

**Supplementary table III. Multivariate regression analysis for BI score ≥60 at discharge**

|  | OR | 95%CI | *P* value |
| --- | --- | --- | --- |
| Age, per year | 0.964 | 0.930-0.999 | 0.043 |
| Cerebral infarction | 2.131 | 0.614-7.398 | 0.233 |
| Diabetes mellitus | 0.494 | 0.036-6.802 | 0.598 |
| Hypoglycemic therapy | 3.716 | 0.273-50.565 | 0.324 |
| Speech therapy | 7.075 | 1.298-38.580 | 0.024 |
| Swallowing therapy | 1.200 | 0.264-5.443 | 0.814 |
| Cognitive therapy | 0.553 | 0.143-2.147 | 0.392 |
| Respiratory therapy | 1.448 | 0.288-7.288 | 0.654 |
| LDL, per mmol/L | 0.986 | 0.506-1.919 | 0.966 |
| WBC, per mmol/L | 1.339 | 1.081-1.659 | 0.008 |
| BI score at admission, per point | 1.139 | 1.090-1.190 | <0.001 |
| Numbers of rehabilitation therapy | 0.648 | 0.287-1.466 | 0.298 |

OR, odds ratio; CI, confidence interval; LDL, low density lipoprotein; WBC, white blood cell; BI, Barthel index.
